# Virtual patient simulation strengthens confidence in clinical conversations among undergraduated nursing students: a randomized controlled trial

**DOI:** 10.1186/s12909-025-08413-y

**Published:** 2025-12-08

**Authors:** Karolina Sörman, Samir El Alaoui, Sophie Mårtensson, Margaretha Larsson, Rajna Knez, Madeleine Ljudvåg, Rebecka Mac, Karin Dahlström, Ylva Elvin Nowak, Uno Fors, Joachim Eckerström

**Affiliations:** 1 Department of Clinical Neuroscience, Centre for Psychiatry Research, Karolinska Institutet & Stockholm Health Care Services, Norra Stationsgatan 69, 7th floor, Stockholm, Region Stockholm 113 64 Sweden; 2 School of Health Sciences, University of Skövde, Skövde, Sweden; 3 Skaraborg Hospital, Skövde, Sweden; 4 Institute of Neuroscience and Physiology, Sahlgrenska Academy, University of Gothenburg, Gothenburg, Sweden; 5 Academic Primary Health Care Centre, Stockholm, Region Stockholm Sweden; 6 Department of Women´s and Children´s Health, Karolinska Institutet, Stockholm, Sweden; 7 Department of Neurobiology, Division of Family Medicine and Primary Care, Care Sciences and Society, Karolinska Institutet, Huddinge, Sweden; 8 Department of Computer and Systems Sciences (DSV), Stockholm University, Stockholm, Sweden; 9 Division of Nursing, Department of Neurobiology, Care Sciences and Society, Karolinska Institutet, Huddinge, Sweden

**Keywords:** Virtual patient, Simulation-based education, Nursing education, Intimate partner violence, Randomized controlled trial

## Abstract

**Background:**

Intimate partner violence (IPV) is a major public health concern, and healthcare staff must be competent in identifying and responding to IPV. However, training opportunities to handle such cases remain limited. This study examined whether integrating a virtual patient (VP) simulation into nursing education improves confidence, self-efficacy, and knowledge in addressing IPV.

**Methods:**

In this randomized controlled trial (RCT), fifty-four nursing students (87% female) were randomly assigned to an intervention group (*n* = 29) receiving a VP module in addition to standard training, or a control group (*n* = 25) receiving standard training only. Students were assessed at baseline and after each educational activity: (1) a web-based module (both groups) (2), VP training for the intervention group while the control group had no corresponding module, and (3) a teacher-led seminar (both groups). Primary outcomes were confidence in asking about IPV, general self-efficacy, self-assessed knowledge, and objective knowledge. Linear mixed-effects models were used to analyse changes over time.

**Results:**

The VP group showed a significantly greater improvement in confidence in asking about IPV (between-group difference + 1.26, 95% CI 0.40–2.15; *p* = 0.010). Objective knowledge scores were consistently higher in the VP group, although the group × time interaction was not significant. General self-efficacy and self-assessed knowledge improved similarly in both groups.

**Conclusions:**

Integrating an interactive VP simulation into a nursing curriculum enhances learners’ confidence in addressing IPV and is associated with higher levels of factual knowledge. VP simulations may represent a scalable complement to conventional teaching for complex and sensitive clinical topics.

**Trial registration:**

ClinicalTrials.gov ID: NCT06706011.

**Supplementary Information:**

The online version contains supplementary material available at 10.1186/s12909-025-08413-y.

## Background

In psychiatry and other medical fields, various of digital methods are used, including simulated environments, mental health apps, chatbots and smart watches for real-time symptom tracking, aiming to improve diagnosis and treatment [1]. This development highlights the need for educational approaches that strengthen digital competence among healthcare professionals and students, such as online courses, virtual patients (VPs) and virtual reality-based training [2].

Intimate partner violence (IPV) is a significant public health issue and the focus of the current study. Internationally, up to one-third of women have been subjected to IPV at some point in their lives [3]. In Sweden, 5% of women experience recurrent IPV [4]. Exposure to IPV is associated with a range of negative physical and psychological health consequences, including gynecological problems, chronic pain, anxiety, depression, and post-traumatic stress disorder [5]. Victims of IPV commonly seek health care without revealing the exposure to violence [6], which underscores the importance of adequate training among health care personnel. Despite this need, several national and international studies have demonstrated that clinicians often find it difficult to ask questions about IPV and to know how to respond when disclosure occur [7]. Given the high prevalence and symptom burden, along with these challenges in clinical practice, IPV is a promising area for development and evaluation of innovative pedagogical methods [8].

High-fidelity simulation has been shown to enhance nursing students’ performance, satisfaction, and self-confidence across various clinical learning contexts [9–12] These studies highlight the value of immersive simulation-based education in strengthening communication and clinical decision-making skills, supporting the integration of virtual patient (VP) simulations as a complementary digital learning approach in nursing education. VPs are particularly suited for practicing the assessment of complex patient profiles, including rare and atypical conditions [2]. VPs are interactive simulations that allow users to practice decision-making, assessment and clinical reasoning in a safe environment. A systematic review of 51 trials with 4696 students compared the effectiveness of VPs with more traditional teaching methods and other forms of digital education. VPs were generally superior to traditional education in improving practical skills (e.g., clinical reasoning and teamwork) and equally effective in improving knowledge, although overall study quality was limited [13]. A systematic review in nursing [14] similarly found that VPs can improve confidence, knowledge, skills, and competence in a safe and controlled environment. More specifically, VPs have been shown to be efficient learning tools in cases involving vulnerable populations [15]. For example, studies using VPs reflecting patients from different ethnic and migrant backgrounds have demonstrated that VP training can foster improved cultural competence among physicians and medical students [16]. VPs can be adapted to various educational contexts and learning levels, including psychiatric nursing [14]. A study of psychiatrists in transcultural psychiatry training demonstrated increased confidence in identifying and evaluating trauma-related diagnoses when using a VP, supporting the role of VPs as a valuable complement to traditional education in complex cases areas [17].

Combining VP interactions with feedback and reflective activities may further facilitate the transfer of learned skills into clinical practice [13, 14]. However, methodological limitations have been noted in prior VP trials. For example, Sahin Karaduman and Basak [14] reported that only four out of ten randomized controlled trials (RCTs) determined sample size by power analysis, and in six studies the sampling method was not specified. Kononowicz, Woodham [13] also highlighted unclear descriptions of randomization methods, and a frequent lack of validated instruments. To advance the field, there is a need for high-quality RCTs using standardized assessments to evaluate the efficacy of VP tools and inform best practices [18]. The present study therefore aimed to evaluate the use of a virtual patient case (VP) as a digital educational tool, and to examine whether its integration into traditional nursing education could enhance nursing students’ [1] confidence in addressing IPV during clinical conversations [2], general self-efficacy [3], self-assessed knowledge, and [4] objective knowledge related to IPV. It was hypothesized that students in the VP group would show greater improvements in confidence, self-efficacy, and knowledge than those in the control group.

## Methods

### Study design and setting

**Fig. 1:**
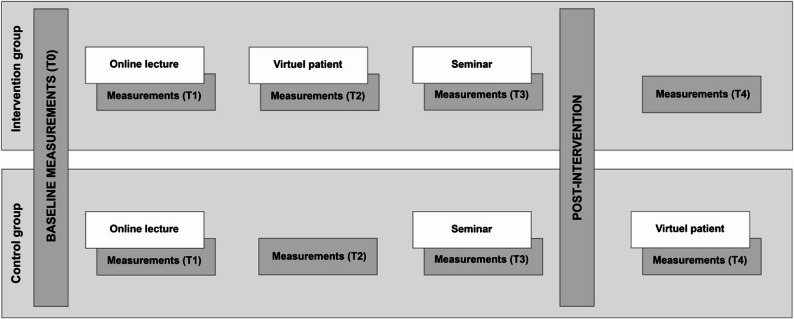
Overview of intervention vs. control group activities and measurement time points

An individually randomised, two-arm, parallel-group superiority trial was conducted during the autumn term of 2024 in the fourth-semester Bachelor of Nursing course at the University of Skövde (Sweden). The trial adhered to CONSORT 2010 guidelines and was prospectively registered on ClinicalTrials.gov (NCT06706011), registration data November 26, 2024. The study was approved by the Swedish Ethical Review Authority (reference number 2023–06687-01), and all participants provided electronic informed consent prior to enrolment. The intervention arm completed an interactive virtual-patient (VP) simulation in addition to the usual curriculum, whereas the control arm received the standard curriculum alone; for ethical reasons, control students were granted access to the VP module after the final follow-up (Fig. 1).

### Randomisation and allocation

**Fig. 2:**
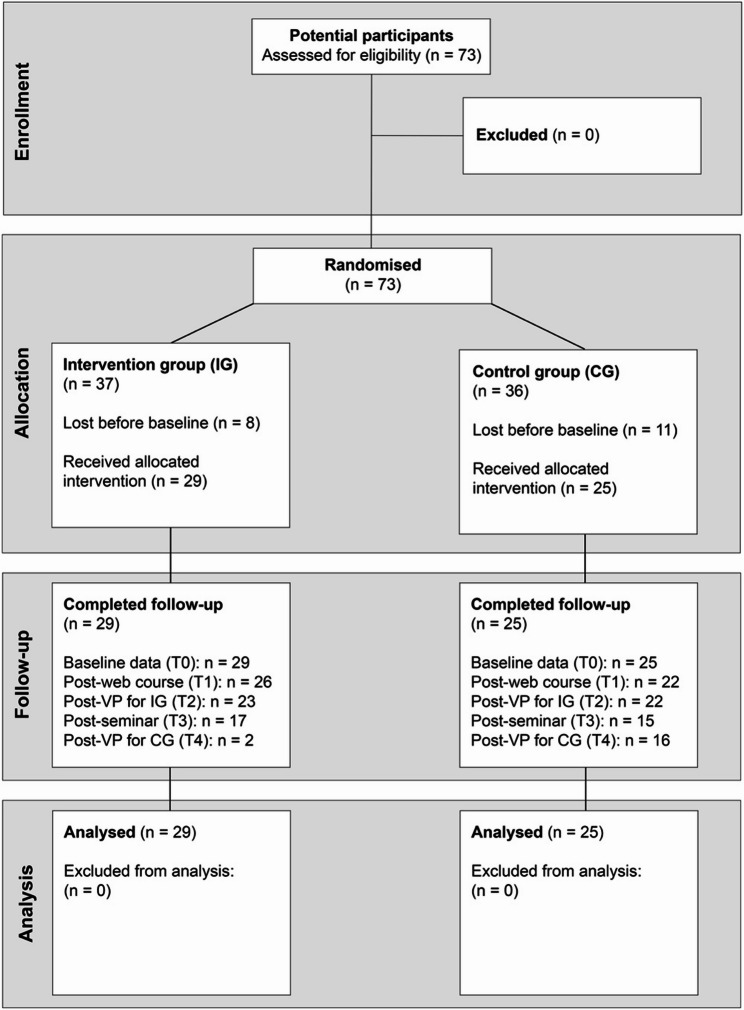
CONSORT 2010 flow diagram showing recruitment, allocation, follow-up and analysis of nursing-student participants. Note. Counts at each time-point reflect students who returned at least one outcome measure (VAS-knowledge, VAS-self-efficacy or GSE). Attrition between time-points resulted primarily from students failing to open subsequent REDCap survey links; no withdrawals were due to adverse events

A 1:1 allocation sequence was generated using the independent web-based randomization tool RANDOM.ORG List Randomizer. The sequence was created a priori by a researcher not involved in enrolment, ensuring allocation concealment until participants were assigned to groups. No blocking or stratification was applied. Participant flow is shown in Fig. 2.

### Participants

Eligible participants were all students enrolled in the fourth semester of the Bachelor of Nursing program at the University of Skövde during autumn 2024. No additional exclusion criteria were applied. In total, 73 students were invited (Fig. 2). Following randomisation, 19 students (8 VP, 11 control) did not complete electronic informed consent and baseline questionnaires in Research Electronic Data Capture (REDCap) and therefore contributed no outcome data; consistent with CONSORT guidelines, they were excluded from all analyses. The analytic cohort comprised 54 students (29 VP, 25 control), representing 74% of those randomised. Post-randomisation attrition occurred before any study procedures or outcome measurements, making bias unlikely. The imbalance between groups (37 vs. 36 planned → 29 vs. 25 analysed) is reported transparently. Baseline demographic characteristics including age, gender, semester, clinical experience and prior experience of IPV inquiry were collected and used as covariates in subsequent analyses to control for potential confounding.

No formal power analysis was performed. The study included all nursing students enrolled in the fourth-semester course during the autumn term of 2024, representing the full population for that period. Consequently, the final sample size (*N* = 54) reflects pragmatic feasibility rather than statistical optimization. The trial was therefore considered exploratory, aimed at assessing feasibility and preliminary educational effects of integrating a virtual patient module into existing coursework.

### Intervention

The intervention group received a three-part interactive educational module on IPV, consisting of (1) a web-based education (2), training with a VP, and (3) a teacher-led follow-up seminar. The control group completed only the web-based education and seminar, without access to the VP simulation during the study period. The intervention was designed to support nursing students’ ability to recognise, address and reflect on IPV-related cases in clinical practice.

#### Web-based education

All participants completed a web-based education focusing on IPV in healthcare. Developed by the Academic Primary Health Care Centre in Region Stockholm, the course aims to equip healthcare professionals with essential knowledge and skills to identify and support individuals exposed to IPV [19]. The training takes approximately 2.5 h to complete and is widely implemented across nursing and medical programmes in Sweden. It covers key topics including IPV prevalence, forms of abuse, health consequences and clinical communication strategies, delivered through textual content, instructional videos and interactive elements such as quizzes and case-based scenarios.

At the University of Skövde, fourth-term nursing students are expected to demonstrate knowledge of the health consequences of IPV as part of their course objectives. Before engaging with the VP simulation, students completed the web-based training and received a short orientation covering the learning objectives, structure and practical instructions for the VP component.

#### Training with a virtual patient

The VP training was developed for psychiatric education with a focus on IPV. The case was designed by members of the current research team (KS, KD, YEN, UF, RM, JE) using the Virtual Case System (VCS) platform from Stockholm University (https://www.casemaker.se/). The scenario featured a 41-year-old fictional patient named Sarah presenting with sleep disturbances, anxiety and depressive symptoms. Pre-recorded video clips with a professional actor provided verbal and realistic nonverbal communication, including eye contact, tone and body language.

The simulation is divided into five main components: (1) background information about the patient case (2), a conversation interface covering key areas such as reason for contact, social context, psychiatric symptoms, and care planning (3), access to completed rating scales including Generalized Anxiety Disorder 7 (GAD-7) [20], Patient Health Questionnaire 9 (PHQ-9) [21], and Alcohol Use Disorders Identification Test (AUDIT) [4, 22] a written assessment section for the student to summarize findings and plan care, and [5] feedback. Feedback consisted of automated responses from the VP character based on the perceived patient experience and expert evaluations. Questions posed by the user were graded as inappropriate (−1), neutral (0) or appropriate (+ 1) across domains such as empathy, open-ended inquiry and information delivery. Each simulation session lasted 30 to 60 min.

#### Teacher led follow-up seminar

Both groups participated in a follow-up seminar facilitated by an experienced educator. These in-person sessions aimed to consolidate learning and encourage reflective discussions. Students were invited to share insights and raise questions based on their training experience. Seminar groups included 6 to 12 participants and lasted approximately 90 min. Common discussion themes included challenges in patient communication, handling emotional complexity and managing conflicting loyalties in IPV situations. The format emphasised peer learning and critical reflection, drawing on Kolb’s Experiential Learning Theory [23].

The seminar facilitator was aware of group allocation because sessions were conducted separately for the VP and control groups within the same course. *To reduce potential bias*,* the facilitator had no role in randomization*,* or outcome assessment.*

### Data collection

Data were collected via REDCap across five time points over a 14-day period. Following baseline assessment (T0), participants completed four additional surveys: T1 immediately after the web-based course (median 2 days post-baseline), T2 after the VP training for intervention participants (median 5 days post-baseline), T3 after the teacher-led seminar for both groups (median 11 days post-baseline), and T4 after VP crossover training for control participants (median 12 days post-baseline). The compressed timeline was intended to minimise exposure to external educational influences and reduce maturation effects, while capturing learning outcomes related to each educational component. The primary analysis was based on T0–T3. Data from T4 were collected to allow the control group eventual access to the VP but were not included in between-group analyses.

### Blinding

All outcomes were self-reported electronically by participants in REDCap; no human assessors were involved. Data analysis was conducted by the study team and was not blinded to group allocation. To reduce potential bias, analyses followed a prespecified plan and all outcomes and model terms were reported according to CONSORT recommendations.

### Outcome measures

Four primary outcomes were assessed to capture both subjective and objective aspects of competence in IPV inquiry.

1. General Self-Efficacy Scale (GSE): The validated 10-item instrument was used, with each item rated on a 4-point Likert scale. Total scores range from 10 to 40, with higher scores indicating greater general self-efficacy.
2. Confidence in asking about IPV (Confidence VAS): Participants rated their confidence in asking patients about IPV on a numeric rating scale ranging from 1 (not confident at all) to 10 (strongly confident). This measure was administered at all assessments.
3. Objective knowledge test: A 15-item test consisting of multiple-choice questions (MCQs) was developed to assess knowledge of IPV and appropriate clinical responses (see Appendix). Each MCQ included between 2 and 9 response options, covering clinical indicators, risk assessment, legal obligations and best practices for supporting patients exposed to IPV. Items were reviewed by subject-matter experts to ensure content validity. Correct responses were assigned positive weights, incorrect responses incurred a penalty, and neutral responses (i.e., options not explicitly correct or incorrect) were scored as 0. Total knowledge scores were computed in SPSS by summing the weighted responses across all items. This weighted scoring system was chosen to capture gradations in knowledge beyond binary correct/incorrect coding. The test was not piloted in a separate cohort, and no separate reliability analysis was performed. The measure was designed for this exploratory trial to assess students’ factual knowledge related to IPV in a standardized manner.
4. Self-assessed knowledge (Knowledge VAS): Participants rated their current knowledge of IPV on a numeric rating scale ranging from 1 (very low knowledge) to 10 (very high knowledge).

### Data analysis

Baseline demographic and clinical characteristics were summarised descriptively by group to assess balance after randomisation. Continuous variables (age, clinical experience, baseline self-efficacy) are reported as means ± standard deviations (SD) and compared between groups using independent-samples t-tests, with Welch’s correction applied when Levene’s test indicated unequal variances. Categorical variables are presented as n (%) and compared using χ² tests, with Fisher’s exact test applied when expected cell counts were < 5. Assumptions of normality and homoscedasticity were evaluated prior to interpreting model results. Normality of residuals was assessed by visual inspection of histograms and Q–Q plots, which indicated approximately normal distributions. Although Shapiro–Wilk tests showed minor deviations (*p* < 0.05) for some variables, these were small and typical for continuous VAS outcomes. Homogeneity of variance between groups was examined using Levene’s tests, all of which were nonsignificant (all *p* > 0.05). No substantial violations of model assumptions were detected. For each outcome, longitudinal changes from baseline to follow-up were analysed using linear mixed-effects models (LMMs) with restricted maximum likelihood estimation (REML). Time (4 levels: baseline, web-based training, VP training, seminar) and Group (control, VP) were entered as fixed effects along with their interaction (Group × Time). A random intercept for each participant was included to account for within-subject correlations, and a variance components covariance structure was specified. Degrees of freedom were estimated using Satterthwaite’s method. The analysis included data from T0–T3; data from T4 (crossover training for control participants) were collected for ethical reasons but not included in between-group analyses. Two sets of models were run for each outcome: “unadjusted” models included Time, Group, and Group × Time effects; “adjusted” models additionally included prior experience in IPV inquiry (coded as binary) to control for baseline exposure. Missing data were handled within the LMM framework, which uses all available observations under the assumption of missing at random. Model fit was evaluated using Akaike’s Information Criterion (AIC), with lower values indicating better fit. Pairwise comparisons among time points were performed with Bonferroni adjustment. Statistical significance was set at two-sided *p* < 0.05. All analyses were conducted in IBM SPSS Statistics (version 29; IBM Corp).

## Results

### Participant characteristics

Baseline characteristics are presented in Table 1. The groups were similar in terms of demographic variables, healthcare experience and prior exposure to IPV inquiry, with no notable differences observed.

**Table 1:** Baseline characteristics of the study participants

Characteristic	Control (*n* = 25)	VP (*n* = 29)	*P* Value
Age, mean (SD), y	28.1 (9.4)	28.5 (6.9)	0.87
Gender, No. (%)			0.31
Female	23 (92.0)	24 (82.8)	
Male	2 (8.0)	5 (17.2)	
Years of experience in healthcare, mean (SD)	6.6 (6.3)	4.1 (4.0)	0.13
Clinical experience in healthcare, No. (%)			0.19
Yes	23 (92.0)	23 (79.3)	
No	2 (8.0)	6 (20.7)	
Clinical field of work, No. (%)			–
Psychiatric care	1 (4.5)	3 (13.0)	
Somatic care	17 (77.3)	17 (73.9)	
Other area	4 (18.2)	3 (13.0)	
Prior experience in IPV inquiry, No. (%)			0.43
Yes	6 (27.3)	4 (17.4)	
No	16 (72.7)	19 (82.6)	
Encountered a situation where IPV inquiry was warranted but not asked, No. (%)			0.23
Yes	3 (12.0)	1 (3.4)	
No	22 (88.0)	28 (96.6)	
Confidence in IPV inquiry (VAS), mean (SD)	6.4 (2.0)	5.6 (1.6)	0.10
General self-efficacy, mean (SD)	31.9 (3.0)	30.5 (3.4)	0.13
Self-assessed knowledge (VAS), mean (SD)	5.2 (1.8)	4.7 (1.9)	0.31
Objective knowledge score, mean (SD)	28.4 (6.1)	30.1 (4.2)	0.21
Educational program of VP training, No. (%)	25 (100)	29 (100)	–
Nursing program (Sjuksköterskeprogrammet)	25 (100)	29 (100)	
University where VP training was completed, No. (%)	25 (100)	29 (100)	–
University of Skövde	25 (100)	29 (100)	
Current semester in education, No. (%)	25 (100)	29 (100)	–
Semester 4	25 (100)	29 (100)	

*P* values for continuous variables are from two-sided t tests; *p* values for categorical variables are from Pearson χ² tests. No differences reached *p* < 0.05. Some variables had partial nonresponse (valid *n* = 22–23 per group)

*Abbreviations*: *IPV* intimate partner violence, *SD* standard deviation, *VAS* visual analogue scale, *VP* virtual patient

### Data completeness

Data completeness was high across variables. At baseline, the four outcome measures (confidence in IPV inquiry, general self-efficacy, self-assessed knowledge, and objective knowledge) and age had complete data for all participants (*N* = 54). Two background variables had item nonresponse: years of experience in healthcare (10/54 missing; 18.5%) and prior experience in IPV inquiry (9/54 missing; 16.7%). The clinical field of work variable was recorded only for participants with clinical experience (valid n: control = 22, VP = 23), reflecting the question’s applicability rather than missing data. Across longitudinal assessments, missing outcome data were minimal (< 4%) and occurred as isolated non-responses at the final time point, with no group-related pattern. All four mixed-effects models included the full analytic cohort (*N* = 54; 216 repeated-measure observations); minor missingness (< 4%) was handled directly by the mixed-model estimation without participant exclusion.

### Longitudinal outcomes

**Fig. 3:**
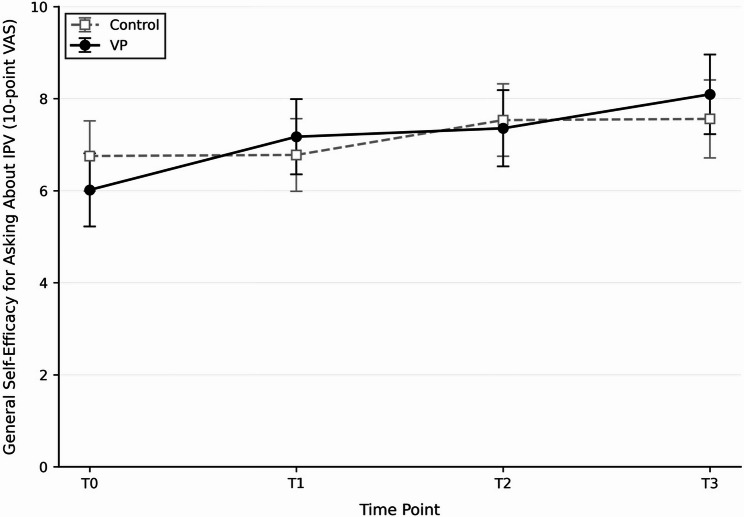
Estimated marginal means with 95% confidence intervals for confidence in asking about intimate partner violence (IPV) across four assessment points: T0 = baseline, T1 = after the web-based module, T2 = after virtual patient (VP) training, and T3 = after the teacher-led seminar. A significant Group × Time interaction (*p* < 0.05) indicated greater improvement in the VP group compared with the control group

Results from adjusted linear mixed-effects models controlling for prior IPV inquiry experience are summarised in Table 2. Model-adjusted group means and 95% confidence intervals at each time point are presented in Table 3 to illustrate descriptive trends across outcomes. General self-efficacy increased significantly over time (*F*[3, 96.96] = 3.98, *p* = 0.010), with no main effect of group (*F*[1, 40.90] = 0.03, *p* = 0.869) and no group × time interaction (*F*[3, 96.99] = 1.09, *p* = 0.356). Students with prior IPV inquiry experience reported higher self-efficacy overall (*F*[1, 41.31] = 6.68, *p* = 0.013). Confidence in asking about IPV showed a significant time effect (*F*[3, 95.23] = 16.94, *p* < 0.001) and a significant group × time interaction (*F*[3, 95.28] = 3.98, *p* = 0.010), indicating greater improvement among VP participants (see Fig. 3). Objective knowledge was significantly higher in the VP group overall by an average of 3.6 points (*F*[1, 35.58] = 6.07, *p* = 0.019), although the group × time interaction was nonsignificant (*p* = 0.230), suggesting parallel improvement across groups. *Self-assessed knowledge* increased significantly over time (F[3,99.01] = 28.39, *p* < 0.001), with no significant group effect (F[1,38.12] = 0.00, *p* = 0.965) and no group × time interaction (F[3,99.02] = 0.73, *p* = 0.535).

**Table 2:** Longitudinal mixed-effects model analyses of educational outcomes

Outcome	Time Effect	Group Effect	Group × Time Interaction	Prior IPV Inquiry Effect	AIC
General Self-Efficacy (GSE)	F(3,96.96) = 3.98, *p* = 0.010	F(1,40.90) = 0.03, *p* = 0.869	F(3,96.995) = 1.09, *p* = 0.356	F(1,41.31) = 6.68, *p* = 0.013	633.44
Confidence in IPV Inquiry (VAS)	F(3,95.23) = 16.94, *p* < 0.001	F(1,38.49) = 0.00, *p* = 0.994	F(3,95.28) = 3.98, *p* = 0.010	F(1,39.16) = 4.00, *p* = 0.052	481.55
Objective Knowledge Score	F(3,95.67) = 4.36, *p* = 0.006	F(1,35.58) = 6.07, *p* = 0.019^a^	F(3, 95.68) = 1.46, *p* = 0.230	F(1, 36.25) = 0.77, *p* = 0.386	880.67
Self-Assessed Knowledge (VAS)	F(3,99.01) = 28.39, *p* < 0.001	F(1,38.12) = 0.00, *p* = 0.965	F(3,99.02) = 0.73, *p* = 0.535	F(1,38.96) = 3.78, *p* = 0.059	502.58

Mixed-effects models contained fixed terms for Group, Time (categorical, four waves), their interaction, and prior experience of IPV inquiry (yes/no). The Time effect reflects overall change across waves collapsed over groups; the Group effect reflects the average difference between VP and control arms; and the Group × Time interaction tests whether trajectories differ between arms. For Objective Knowledge Score, the main Group effect was significant (*F* = 6.07, *p* = 0.019) while the interaction was not, indicating that both groups improved at a similar rate but the VP arm maintained an ≈ 3.6-point advantage at every wave

*Abbreviations*: *AIC* Akaike information criterion, *GSE* general self-efficacy, *IPV* intimate partner violence, *NS* = non-significant (*p* >.05), *VAS* visual analogue scale, *VP* virtual patient

**Table 3:** Changes in primary outcomes from baseline to final assessment, adjusted for prior IPV inquiry (model-adjusted means with 95% confidence intervals)

Outcome	Baseline, Control Mean (95% CI)	Final, Control Mean (95% CI)	Change in Control	Baseline, VP Mean (95% CI)	Final, VP Mean (95% CI)	Change in VP	Between-Group Difference in Change (95% CI)	*P* Value
General Self-Efficacy (GSE)	32.32 (28.34, 36.29)	32.68 (28.65, 36.70)	+ 0.36	31.80 (27.80, 35.80)	33.00 (28.96, 37.05)	+ 1.20	+ 0.84 (–1.20, 2.88)	0.356
Confidence in IPV Inquiry (VAS)	6.75 (4.84, 8.67)	7.56 (5.61, 9.51)	+ 0.81	6.02 (4.09, 7.94)	8.09 (6.14, 10.05)	+ 2.07	+ 1.26 (0.40, 2.15)	0.010
Objective Knowledge Score	29.00 (22.19, 35.81)	31.96 (24.96, 38.96)	+ 2.96	31.29 (24.45, 38.13)	35.10 (28.10, 42.10)	+ 3.81	+ 0.85 (–2.12, 3.82)	0.230
Self-Assessed Knowledge (VAS)	5.60 (3.82, 7.39)	7.58 (5.74, 9.47)	+ 1.98	5.24 (3.44, 7.03)	7.63 (5.79, 9.47)	+ 2.39	+ 0.41 (–1.31, 2.13)	0.535

Values represent estimated marginal means from linear mixed-effects models including fixed terms for Group, Time, their interaction (Group × Time), and prior experience of IPV inquiry (covariate). “Change” indicates the within-group difference (Final − Baseline EMM). The between-group difference in change (Δ VP − Δ Control) corresponds to the Group × Time interaction; its *p* value tests whether improvement differed between groups. Bonferroni-adjusted pairwise contrasts produced identical conclusions

*Abbreviations*: *CI* confidence interval, *IPV* intimate partner violence, *VP* virtual patient, *VAS* visual analogue scale

### Between-group differences in change

From baseline to final assessment (Table 3), the VP group showed significantly greater improvement in confidence in IPV inquiry compared with controls (between-group difference: +1.26 points, 95% CI 0.40–2.15, *p* = 0.010). On the 10-point scale, this corresponded to an average gain of 2.07 points in the VP group (6.02 to 8.09) versus 0.81 points among controls (6.75 to 7.56), indicating a substantially larger absolute improvement in the intervention group. No significant between-group differences in change were observed for general self-efficacy (+ 0.84 points, 95% CI − 1.20 to 2.88, *p* = 0.356), objective knowledge (+ 0.85 points, 95% CI − 2.12 to 3.82, *p* = 0.230), or self-assessed knowledge (+ 0.41 points, 95% CI − 1.31 to 2.13, *p* = 0.535). Nevertheless, objective knowledge scores remained consistently higher in the VP group across assessments (mean = 35.10 vs. 31.96 at follow-up), suggesting a favorable but nonsignificant trend toward improved learning outcomes.

## Discussion

In this randomized controlled trial among nursing students, the key finding was that the VP intervention produced a significantly greater improvement in confidence in ability to ask questions about IPV and maintained learners’ higher levels of factual knowledge. Both the control and VP groups demonstrated significant improvements from pre- to post-assessment across several educational outcomes. Baseline characteristics were similar between groups, but differences emerged over the study period. Both groups improved in general self-efficacy and self-assessed knowledge, with no significant differences between arms, suggesting that the standard curriculum is effective in strengthening students’ general perceptions of capability and subjective knowledge. Both groups improved over time regarding objective knowledge, but the VP group consistently scored higher across all assessments, averaging 3.62 points above controls.

These results build on our earlier evaluation of the VP, in which 62 nursing students completed the module and considered it user-friendly, intuitive, and beneficial for learning about IPV and practicing patient dialogues [24]. The present RCT extends this work with stronger methodology, addressing calls in recent reviews for more rigorous designs in the field [13, 14], emphasizing that the quality of evidence in RCT designs is low to moderate. Gaining an increased confidence in addressing IPV could have to do with several aspects specific for the VP platform such as realism of the scenario (e.g., both in terms of background info of the patient and the assessment scheme) but also being a safe practice environment.

A significant group × time interaction underscores the unique impact of the VP module in enhancing clinical confidence, a critical barrier identified in IPV-related care. The significant group × time interaction for confidence in IPV inquiry highlights the added value of VP training for enhancing clinical confidence, a barrier frequently described in IPV-related care. These findings resonate with suggestions that students in health professions need efficient ways to practice communication skills in sensitive areas [25]. VP training may provide additional support for factual learning, particularly through integrated assessment components and structured expert feedback. Prior reviews have emphasised that VP tools may be especially effective when knowledge acquisition is linked with problem-solving and applied clinical reasoning [13, 26]. Prior IPV inquiry experience predicted higher general self-efficacy irrespective of group, underscoring the potential importance of experiential learning. Students who had already engaged in IPV-related inquiry may have felt more confident with difficult clinical conversations, potentially complementing VP training.

### Limitations

The relatively small sample size (*N* = 54) and single-site design limit the generalizability of the findings, as participants represented a homogeneous cohort of nursing students with similar clinical backgrounds. The majority were female, reflecting the demographics of the nursing program, which may reduce applicability to other populations such as students from different disciplines or academic levels. Nineteen invited students did not complete informed consent or baseline questionnaires and were therefore excluded prior to participation. Because this attrition occurred before randomization and any study procedures, the risk of selection bias is likely low. Replication in larger and more diverse cohorts across multiple institutions is warranted to strengthen external validity.The follow up-time was short (11 days in total), future research should include investigations of longer retention periods. Although prior IPV inquiry experience was included as a covariate, other factors such as age and broader clinical experience were not considered and may have influenced the results. Another limitation is that the control group received one fewer educational component than the intervention group; however, apart from the VP module, the tracks were identical, which strengthens comparability. Reliance on self-reported measures may also have introduced bias, as participants could have over- or underestimated their abilities. While the objective knowledge test provided a structured assessment, it was study-specific and not externally validated. Incorporating performance-based evaluations and validated instruments in future research would improve robustness.

## Conclusions

This study contributes to the research field of VPs, given that it is one of the few RCTs on VP use in IPV educations. Adding a virtual patient (VP) simulation to undergraduated nursing education significantly enhanced students’ confidence in asking about intimate partner violence (IPV) and sustained higher levels of factual knowledge. While no additional effects were observed for general self-efficacy or self-assessed knowledge, the results highlight the value of VP modules as a complement to traditional teaching in preparing students for challenging clinical conversations. Future research should replicate these findings in larger and more diverse cohorts, assess long-term outcomes in clinical practice and patient care, and explore the applicability of VP training in other sensitive areas of healthcare education, such as suicide risk assessment, communication related to discrimination or stigma, and transcultural aspects of patient care.

## Supplementary Information

Supplementary Material 1.

## Data Availability

Data are available from the corresponding author on reasonable request.

## Abbreviations

AIC: Akaike’s Information Criterion
AUDIT: Alcohol Use Disorders Identification Test
CI: Confidence Intervals
CG: Control Group
GAD-7: Generalized Anxiety Disorder 7
GSE: General Self-Efficacy Scale
IG: Intervention Group
IPV: Intimate partner violence
LMMs: Linear Mixed-effects Models
MCQs: Multiple-choice questions
PHQ-9: Patient Health Questionnaire 9
RCT: Randomized controlled trials
REDCap: Research Electronic Data Capture
REML: Restricted Maximum Likelihood Estimation
SD: Standard Deviation
SPSS: Statistical Package for the Social Sciences
VAS: Visual Analogue Scale
VCS: Virtual Case System
VP: Virtual Patient
