# Supplementary material for: Virtual patient simulation strengthens confidence in clinical conversations among undergraduated nursing students: a randomized controlled trial
Source: BMC Med Educ. 2025 Dec 8;25:1690. doi: 10.1186/s12909-025-08413-y (PMC12690956; doi:10.1186/s12909-025-08413-y)
Supplement: Supplementary file 1 — Supplementary Material 1. [file 12909_2025_8413_MOESM1_ESM.docx]

# Supplementary material

# Knowledge Assessment Questionnaire on Intimate Partner Violence (IPV)

#### Scoring Guidelines:

- Each question has a maximum of **3 points** for correct responses.
- Incorrect answers result in a deduction of **0.5 points**.
- "None of the above" and "Don't know" responses are treated as missing values and assigned **0 points**.

### Knowledge Assessment on Intimate Partner Violence (IPV)

#### 1. Which of the following statements align with your understanding of intimate partner violence?

1. Intimate partner violence is so uncommon that healthcare professionals rarely encounter it.
   -0.5 points
2. The type of violence healthcare professionals typically encounter is physical violence.
   -0.5 points
3. It is important to encourage a person subjected to violence to leave the relationship.
   -0.5 points
4. Women are more frequently subjected to controlling behaviors than men by their partners.
   -0.5 points
5. A person subjected to violence usually returns to their abuser several times before leaving.
   +3 points
6. Psychological violence in close relationships is not as harmful to health as physical violence in close relationships.
   -0.5 points
7. None of the above.
   0 points (Missing value)
8. Don't know.
   0 points (Missing value)

#### 2. Which of the following sign/s would make you suspect that a patient has been/is being subjected to intimate partner violence?

1. Physical injuries to the body.
   0 points (Neutral response)
2. Mental health issues.
   0 points (Neutral response)
3. Chronic or long-term unexplained somatic pain.
   +0.5 points
4. Injuries to the body that do not match the explanations the patient provides regarding how they occurred.
   +0.5 points
5. An accompanying person who refuses to leave the patient alone.
   +0.5 points
6. Fear of a physical examination.
   +0.5 points
7. Very talkative and demanding during interactions.
   -0.5 points
8. Difficulties expressing themselves.
   -0.5 points
9. None of the above.
   0 points (Missing value)
10. Don't know.
    0 points (Missing value)

#### 3. Which of the following statement/s suggest that a patient has been subjected to/is being subjected to physical, psychological, or sexual violence in a close relationship?

1. When their partner curses at them during arguments.
   -0.5 points
2. When they are pushed, kicked, and have their hair pulled by their adult son.
   +0.6 points
3. When they are forced to watch pornography with their partner.
   +0.6 points
4. When they are regularly insulted by their partner about their clothing, weight, and how they manage household tasks.
   +0.6 points
5. When their ex-partner sends multiple text messages each day asking them to come back and threatens to commit suicide.
   +0.6 points
6. When their parents prevent them from seeing friends, having a bank account, or having a boyfriend/girlfriend.
   +0.6 points
7. When their parents state that as long as they live at home, there are rules to follow.
   -0.5 points
8. None of the above.
   0 points (Missing value)
9. Don't know.
   0 points (Missing value)

#### 4. What is important when meeting a patient who has been subjected to/is being subjected to intimate partner violence?

1. Avoid time constraints.
   +0.6 points
2. Listen empathetically and build trust.
   +0.6 points
3. Encourage the patient to leave their abusive partner.
   -0.5 points
4. Discuss violence using concrete terms.
   +0.6 points
5. Help the patient get in touch with social services if the patient wants it.
   +0.6 points (Crucial response)
6. Inform the patient that medical records will be coded under hidden terms, making them invisible in the national health records.
   +0.6 points (Crucial response)
7. None of the above.
   0 points (Missing value)
8. Don't know.
   0 points (Missing value)

#### 5. Why should healthcare professionals ask about exposure to intimate partner violence?

1. Because people subjected to violence may be victims of crime.
   -0.5 points
2. Because the law requires healthcare professionals to ask.
   -0.5 points
3. Because there is a strong connection between poor health and exposure to intimate partner violence.
   +1.5 points
4. Because there may be minor children in the family.
   -0.5 points
5. Because the guidelines of the National Board of Health and Welfare recommend it.
   +1.5 points
6. None of the above.
   0 points (Missing value)
7. Don't know.
   0 points (Missing value)

#### 6. How do you ask a patient if they have been/are being subjected to intimate partner violence?

1. By explaining what intimate partner violence is.
   +0.75 points (Most important response)
2. By asking direct questions.
   +0.75 points
3. By verbally asking pre-determined questions.
   0 points (Neutral response)
4. By avoiding the term 'violence' and trying to use euphemisms.
   -0.5 points
5. By asking the patient to fill out a written questionnaire.
   0 points (Neutral response)
6. None of the above.
   0 points (Missing value)
7. Don't know.
   0 points (Missing value)

#### 7. What do you do if a patient tells you that they are subjected to intimate partner violence?

1. Inform them that everyone has the right to live a life free of violence.
   +0.42 points
2. Inform them that it is harmful for children to witness violence.
   +0.42 points
3. Inform them of their right to support and help from healthcare and social services.
   +0.42 points
4. Encourage the patient to immediately leave the relationship.
   -0.5 points
5. Inform them that the medical record can be protected.
   +0.42 points
6. File a report of concern to social services (if there are children in the home).
   +0.42 points
7. Help the patient get in touch with social services if the patient consents.
   +0.42 points
8. Schedule a follow-up or refer to a psychologist/counselor.
   0 points (Neutral response)
9. Contact the police.
   0 points (Neutral response)
10. None of the above.
    0 points (Missing value)
11. Don't know.
    0 points (Missing value)

#### 8. Which actions are you required to take when a patient reports having been subjected to/is currently being subjected to intimate partner violence?

1. Help the patient get in touch with social services.
   +0.75 points
2. File a report of concern to social services (if there are children in the home).
   +0.75 points
3. Inform them about the possibilities of healthcare support and assistance.
   +0.75 points
4. Document symptoms, injuries, and interventions under hidden search terms.
   +0.75 points
5. Offer a follow-up with yourself or someone else in healthcare.
   0 points (Neutral response)
6. Refer to psychosocial support.
   0 points (Neutral response)
7. Refer to women’s shelters.
   0 points (Neutral response)
8. Refer to centers for relationship violence or equivalent services.
   0 points (Neutral response)
9. Refer to the police.
   0 points (Neutral response)
10. None of the above.
    0 points (Missing value)
11. Don't know.
    0 points (Missing value)

#### 9. Which authorities and/or organizations is healthcare obliged to collaborate with in cases of intimate partner violence?

1. Other healthcare organizations.
   -0.5 points
2. Social services in the municipality.
   +3 points
3. The police.
   0 points (Neutral response)
4. The Prosecutor’s Office.
   -0.5 points
5. Women’s shelters.
   0 points (Neutral response)
6. Victim support services.
   -0.5 points
7. Internal collaboration within the organization.
   -0.5 points
8. None of the above.
   0 points (Missing value)
9. Don't know.
   0 points (Missing value)

#### 10. Apart from obligations, which authorities and/or organizations can healthcare collaborate with in cases of intimate partner violence?

1. Other healthcare organizations.
   +0.75 points
2. The police.
   +0.75 points
3. The Prosecutor’s Office.
   -0.5 points
4. Women’s shelters.
   +0.75 points
5. Victim support services.
   -0.5 points
6. Internal collaboration within the organization.
   +0.75 points
7. None of the above.
   0 points (Missing value)
8. Don't know.
   0 points (Missing value)

#### 11. Do you ask if there are children under 18 in the family when you suspect that a patient is subjected to intimate partner violence?

1. I ask.
   +3 points (Most important)
2. I don’t ask.
   -0.5 points
3. It depends.
   -0.5 points
4. Don't know.
   0 points (Missing value)

#### 12. What should you do if you suspect a patient is subjected to violence and you find out there are children under 18 in the home?

1. File a report of concern to social services.
   +3 points (Most important)
2. Contact social services for advice.
   0 points (Neutral response)
3. Have a serious conversation with the child's guardian or ensure that a colleague does so.
   -0.5 points
4. File a police report.
   0 points (Neutral response)
5. Ask how the child/children are doing.
   0 points (Neutral response)
6. Ask a colleague, supervisor, or my manager what to do.
   0 points (Neutral response)
7. None of the above.
   -0.5 points
8. Don't know.
   0 points (Missing value)

#### 13. Do you ask if there are children under 18 in the family if the patient tells you they are subjected to intimate partner violence?

1. I ask.
   +3 points
2. I don’t ask.
   -0.5 points
3. It depends.
   -0.5 points
4. Don't know.
   0 points (Missing value)

#### 14. What are you required to do if a patient tells you they are subjected to intimate partner violence and there are children under 18 in the home?

1. File a report of concern to social services.
   +3 points
2. Contact social services for advice.
   -0.5 points
3. Have a serious conversation with the parents or ensure that a colleague does so.
   -0.5 points
4. File a police report.
   0 points (Neutral response)
5. Ask how the child/children are doing.
   -0.5 points
6. Ask a colleague, supervisor, or manager what to do.
   -0.5 points
7. None of the above.
   0 points (Missing value)
8. Don't know.
   0 points (Missing value)

#### 15. What are you required to say to the patient subjected to intimate partner violence, if it is revealed that there are children in the home?

1. That you are concerned for the child.
   0 points (Neutral response)
2. That it may be illegal for children to witness violence between adults in the home.
   0 points (Neutral response)
3. That it is harmful for children to witness violence between adults in the home.
   +3 points
4. I ask what help and support the patient wants.
   0 points (Neutral response)
5. None of the above.
   0 points (Missing value)
6. Don't know.
   0 points (Missing value)

### Total Possible Score Range:

- **Maximum score:** 45 points (15 questions × 3 points per question).
- **Minimum score:** It is possible to receive a negative score due to incorrect answers (-0.5 per wrong answer), but the total could also remain at **0 points** if only missing values or neutral responses are selected throughout.
